# Value of three-dimensional endoanal ultrasound scan (3D-EAUS) in preoperative assessment of fistula-in-ano

**DOI:** 10.1186/s13104-019-4098-2

**Published:** 2019-01-29

**Authors:** Isuru Sampath Almeida, Umesh Jayarajah, Dakshitha Praneeth Wickramasinghe, Dharmabandhu Nandadeva Samarasekera

**Affiliations:** Department of Surgery, Faculty of Medicine, University of Colombo, Colombo, Sri Lanka

**Keywords:** Fistula-in-ano, 3D endoanal ultrasound scan, Pre-operative assessment

## Abstract

**Objective:**

The aim of this study was to determine the accuracy of three-dimensional endoanal ultrasound scan (3D-EAUS) in the pre-operative assessment of fistula-in-ano in identifying the fistula tract and comparing with findings at surgery in a South Asian cohort. A retrospective analysis of 87 patients with suspected fistula-in-ano who underwent pre-operative 3D-EAUS between January 2009 and January 2016 was carried out. All patients subsequently had surgical exploration under anaesthesia (EUA), irrespective of 3D-EAUS findings. The 3D-EAUS results were compared with the surgical findings to determine the accuracy of 3D-EAUS.

**Results:**

A total of 86 (98.9%) patients (male = 75) were subsequently shown to have a fistula at surgical exploration and of them, 3D-EAUS detected a fistula in 79 (92%) patients. In this cohort, 3D-EAUS correctly predicted the surgical findings in (n = 61, 70.9%) patients with the highest accuracy being for transphincteric fistulae (87.1%). However, the overall concordance in our study was low with a kappa coefficient of 0.318. Additional findings such as sphincter defects were detected by the 3D-EAUS in 37 patients (internal sphincter defects-21, external sphincter defects-7, both-9) which were not evident at EUA. Therefore, 3D-EAUS had a good accuracy in selected types of fistulae and particularly useful in identifying sphincter defects before surgery.

**Electronic supplementary material:**

The online version of this article (10.1186/s13104-019-4098-2) contains supplementary material, which is available to authorized users.

## Introduction

Fistula-in-ano is a common condition encountered in surgical practice. The goal of the surgery is to eradicate the fistula tract while preserving anal continence [1]. Therefore, accurate preoperative assessment of the anatomy of primary tract, secondary extensions and the internal opening is necessary to achieve optimal surgical results [2, 3].

Numerous methods have been implicated in identifying fistula characteristics. Visual inspection of the perianal area and digital rectal examination under anaesthesia are basic diagnostic methods. However, these methods may fail to identify complex fistulae or localise the internal opening. Though Goodsall’s rule and fistulography have been helpful in locating the internal opening, it varies in accuracy [4, 5].

At present three-dimensional endoanal ultrasonography (3D-EAUS) has become an established technique for imaging the rectum and the anal canal [6]. It provides a detailed multiplanar reconstruction of the anal canal which is expected to be helpful in tracing the tract and the internal opening and in the diagnosis of anorectal pathologies [3, 7]. 3D-EAUS is particularly useful in high fistulae with anal sphincter involvement, especially if there are additional extensions and associated pararectal cavities [8].

There is limited data available on the clinical usefulness and accuracy of 3D-EAUS done in South Asia [9]. Furthermore, the anatomy of the anal sphincters in South Asians are somewhat variable compared to other Asian and Western populations [9]. A study in South Asian females has found that the mean thicknesses of both the internal and external anal sphincters were less than other Asian women. The internal anal sphincter at the mid-sphincter level and the external anal sphincter at the lower sphincter level were thicker posteriorly, while the external anal sphincter at the mid-sphincter level was thicker anteriorly [9]. Therefore an attempt was made to analyse the effectiveness of 3D-EAUS in a cohort of the South Asian population. The purpose of this study was to evaluate the accuracy of 3D-EAUS in pre-operative assessment of anal fistulae in the identification of the fistula tract and compare with findings at surgery.

## Main text

### Patients and clinical evaluation

This is a retrospective analysis of a cohort of 87 patients with suspected anal fistula who underwent pre-operative 3D-EAUS between January 2009 and January 2016. These patients were preoperatively evaluated with a clinical history, digital rectal examination followed by 3D-EAUS without hydrogen peroxide enhancement and subsequently underwent examination under anaesthesia (EUA) and surgery. Those who presented with a history of perianal sepsis and non-healing perianal discharge were suspected to have a fistula. Patients with deeper fistula tracts with sphincter involvement underwent 3D-EAUS. Only those with recurrent multiple fistula tracts underwent an magnetic resonance imaging (MRI) due to limited resources. Those with superficial tracts without any clinical evidence of sphincter involvement did not undergo 3D EAUS. Patients who defaulted the surgical evaluation after the pre-operative 3D-EAUS were excluded from the study.

### 3D-EAUS

**Fig. 1:**
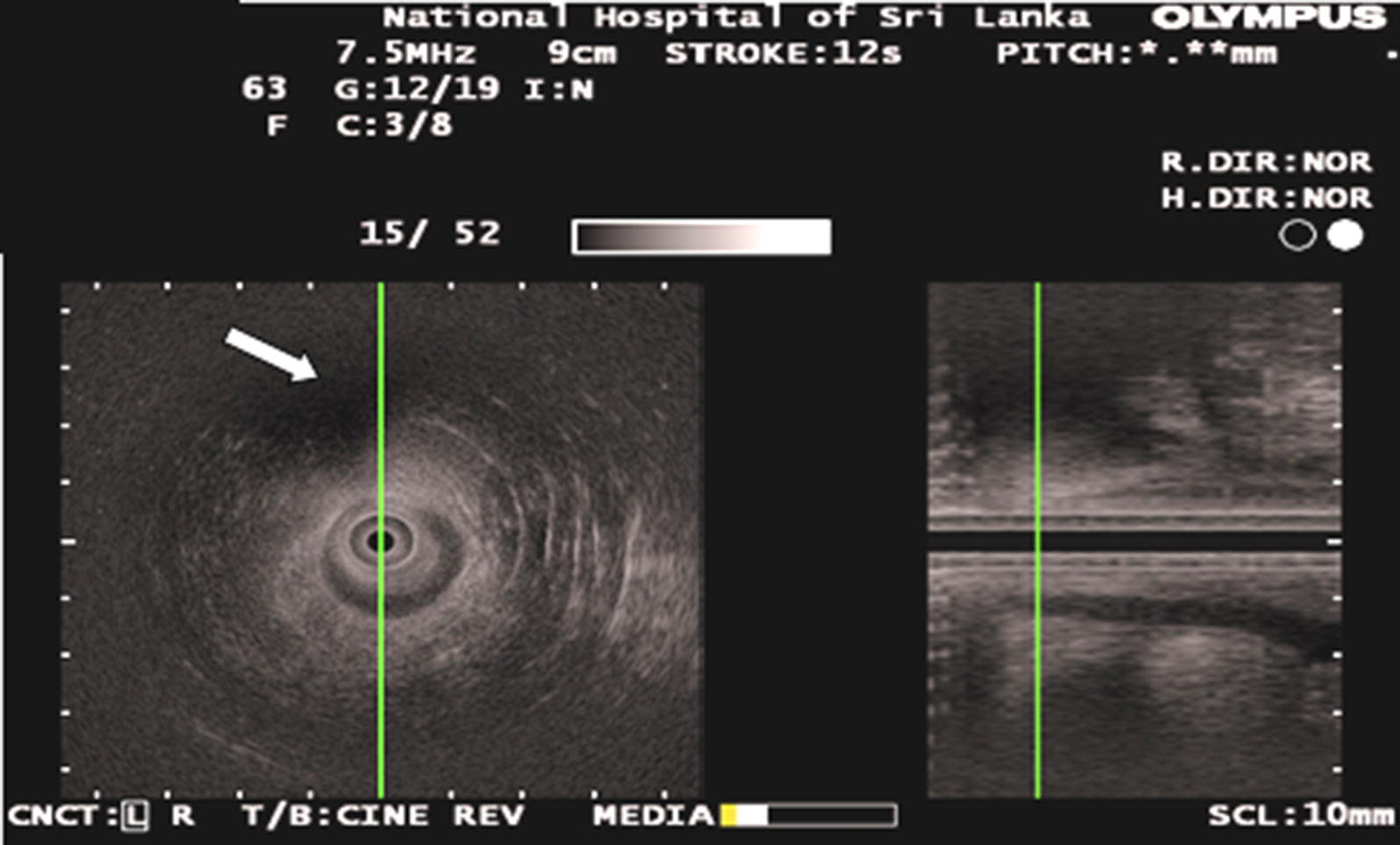
Transverse and sagittal anal endosonogram obtained with a 7.5 MHz transducer at the middle anal canal level shows a transphincteric track (arrow)

All the 3D-EAUS were done by an experienced colorectal surgeon or a senior surgical trainee under direct supervision. Both were experienced in this technique and had performed over 100 procedures before. The examination was performed with a 7.5 MHz, 360^0^, rotating endoprobe (type RU-75M-R1, Olympus^®^) that had a 150 mm length of insertion section. The transducer was covered with a hard sonolucent plastic cone with a 12 mm outer diameter. This transducer was introduced into the anal canal with the patient in left lateral position. 3D images were obtained by an inbuilt function in the system. Fistulae were visualised as hypoechoic tracts. Specific features like, the site of internal opening, level of the radial tract, the relation of tracts to anal sphincters and site of fluid collections/pararectal cavities were obtained. All scans were deemed technically satisfactory and no patient was reinvestigated (Fig. 1).

### Surgical evaluation

Examination under anaesthesia with the patient placed in lithotomy position was carried out by a single colorectal surgeon. The primary opening was located by using conventional fistula probe and/or hydrogen peroxide. The site of external and internal opening(s), the number of fistula tracts, fluid collections were identified and recorded in a standard diagram (Additional file 1: Annexure S1). Exploration under anaesthesia, and laying open and/or placement of setons to fistula tracts were performed as deemed necessary. Those with fistulae with sphincter involvement without associated large collection or pararectal cavities underwent staged seton fistulotomy (n = 78). Those with large cavities were tagged with a draining seton and the cavity was irrigated with an antiseptic solution using an irrigation catheter until the cavity had contracted before performing seton fistulotomy as the definitive procedure (n = 9) [10].

### Statistical analysis

Univariate analysis was done and results of categorical variables were expressed as frequencies and proportions while continuous variables were expressed using means ± standard deviations. The accuracy of 3D-EAUS in detecting and classifying components of a given fistula was compared with the surgical findings as the reference standard. The concordance rate and Kappa coefficient (degree of non-random agreement between different measurements of the same variable) were calculated. The Kappa coefficient varies between − 1 and 1, considering: k = − 1, agreement due to chance; k < 0.2, poor agreement; k = 0.2–0.4, low; k = 0.4–0.6, moderate; k = 0.6–0.8, good; k = 0.8–1, very good.

### Results

3D-EAUS was carried out on 87 patients, which included 76 (87.36%) males and 11 (12.64%) females. The mean age of the population was 39 (SD ± 12.35, range 18–75) years. Most of the patients (n = 86) had a fistula secondary to a cryptoglandular disease and one patient had tuberculosis.

Assessment with 3D-EAUS showed a primary fistula in 80 (92.0%) patients [transphincteric fistulae 66 (75.9%), intersphincteric fistulae 13 (14.9%), extrasphincteric fistula 1 (1.1%)]. Thus, a primary fistula tract was not detected in 7 (8.0%) patients with the use of 3D-EAUS whereas subsequent examination under anaesthesia showed evidence of a fistula tract.

Twenty-one (24.1%) patients were found to have fluid collections, two (2.3%) had secondary extensions apart from the primary fistula tract and one (1.1%) patient had a horseshoe tract during the 3D-EAUS. All collections were deep seated in the ischiorectal fossa. In addition, fistula associated anal sphincter defects were seen in 37 (42.5%) patients, which included 7 (8.1%) patients with external anal sphincter defects, 21 (24.1%) patients with internal anal sphincter defects and 9 (10.3%) patients with defects in both anal sphincters. Anal sphincter defect is the atrophy or disruption of the anal sphincters which is visualised as a discontinuity of the normal echogenicity in the ultrasonography [11].

### Operative assessment

Eighty-six patients examined were subsequently shown to have a fistula tract at surgical exploration and one patient had a sinus tract. Examination under anaesthesia (EUA) revealed 62 (71.3%) transphincteric fistulae, 18 (20.7%) intersphincteric fistulae, 3 (3.4%) suprasphincteric fistulae, 3 (3.4%) superficial fistulae and 1 (1.2%) sinus tract. Apart from the anatomy of the fistula tract, other information such as fluid collections 16 (18.4%), secondary extensions 7 (8.0%), superficial abscess 4 (4.6%) and horseshoe tracts 2 (2.3%) were detected during the EUA.

### Overall accuracy

The accuracy of 3D-EAUS in relation to the surgical findings with respect to correct identification of the primary tract was 70.9% (n = 61/86). The highest concordance was seen for transphincteric fistulae (87.1%). However, there was a significant difference between the overall findings of 3D-EAUS and EUA (p = 0.005). The overall concordance in our study was low with a kappa coefficient of 0.318. The examination under anaesthesia detected 7 more fistulae which were not detected by preoperative 3D-EAUS. These include, 3 transphincteric and intersphincteric fistulae each with one superficial fistula. Table 1 describes surgical and 3D-EAUS findings with respect to identification of the primary tract. The overall accuracy of 3D-EAUS in detecting secondary extensions associated fluid collections and horseshoe tracts are shown in Table 2.

**Table 1:** Results of 3D-EAUS findings in the identification of the primary fistula tract

3D-EAUS	Surgical findings				
	Transphincteric (*n *= 62)	Intersphincteric (*n *= 18)	Suprasphincteric (*n *= 3)	Superficial (*n *= 3)	Sinus tract (*n *= 1)
Transphincteric	54 (87.1%)	8 (44.4%)	2 (66.7%)	1 (33.3%)	1 (100%)
Intersphincteric	4 (6.5%)	7 (38.9%)	1 (33.3%)	1 (33.3%)	0
Extrasphincteric	1 (1.6%)	0	0	0	0
No tract	3 (4.8%)	3 (16.7%)	0	1 (33.3%)	0

**Table 2:** Results of 3D-EAUS findings in classification of secondary extensions, fluid collections and horseshoe tracts

Feature examined	No. of patients correctly classified in 3D-EAUS
Primary tracks (n = 86)	61 (70.9%)
Fluid collections (n = 16)	9 (56.3%)
Extensions (n = 7)	1 (14.3%)
Superficial abscess (n = 4)	0 (0%)
Horseshoe tracts (n = 2)	1 (50%)

### Discussion

Fistula in ano is a challenging surgical problem due to its recurrence and post-operative complications. Infection of the anal glands and crypts is thought to be the cause of subsequent fistula formation. It usually begins as an abscess and develops into a fistula in 60% of cases [12].

Many imaging modalities have been implicated throughout the past with the aim of revealing hidden tracts and to define the relationship of the fistula to the anal sphincters. This plays an important role in planning the operative approach in order to achieve a better surgical outcome. Over the last two decades, EAUS has been a useful tool which demonstrates features accurately in assessing fistula characteristics [6]. EAUS is a rapid, simple and well-tolerated technique. It also provides some valuable information about the anatomy of the anal sphincters. However, EAUS still has some limitations in assessing deep seated cavities and requires experience.

When comparing with other studies done overseas, the results of our study has shown a disparity between the West and South Asia related to fistula characteristics detected with 3D-EAUS. Other similar studies have shown that the accuracy of 3D-EAUS compared with surgery in the identification of the primary tract and abscesses has been in the range of 81–94% and 67–96% respectively [3, 7, 13, 14]. However, in our study 3D-EAUS correctly predicted the primary tract in 70.9% and fluid collections or pararectal cavities in 56.3% of patients. The overall concordance as indicated by kappa coefficient was low in our study. Such a disparity could be related to differences in the complexities of fistulae, the use of variable ultrasound equipment and may also be due to the variations in the anatomy of the anal sphincters. Furthermore, in those studies, the concordance was highest for transphincteric fistulae similar to our study [3, 14].

Several previous studies have denoted surgery as the reference standard [3, 7, 13, 14]. However, the use of surgery as the reference standard may not always be accurate as studies have shown that EAUS is able to identify fistula tracts which are not seen on surgical exploration [15, 16]. In our study, we found one patient with a sinus tract at the surgical exploration which was initially identified as a transphincteric fistula on 3D-EAUS. We used surgical findings as the reference standard as other modes of imaging such as pelvic MRI was not routinely available in our unit. Seven patients with no evidence of fistula in 3D EAUS were shown to have a fistula during EUA. This is probably because the fistula tract was closed during the 3D-EAUS and was difficult to visualise. Those tracts may have opened up with probing and hydrogen peroxide instillation during the surgical exploration.

EAUS with hydrogen peroxide enhancement has been done to improve the diagnostic accuracy of the standard EAUS [17]. This method can thus be helpful in identifying tracts that had not been observed at the standard EAUS examination. Several studies have shown significantly superior results with enhancement, compared to unenhanced EAUS [15, 18, 19]. However, Kim et al. [3] showed no statistically significant difference between 3D-EAUS and hydrogen enhanced 3D-EAUS with respect to classifying primary tracts, internal opening and secondary extensions. Furthermore, a systemic review done by Gianpiero et al. [7] showed results similar to Kim et al. A study by Gordon et al. [20] suggested the use of enhancement technique would be beneficial in difficult cases. Thus a selective 3D-EAUS with enhancement in difficult cases may be a useful option. MRI is regarded as the “gold standard” for pre-operative assessment of anal fistula [21, 22] and stated as being superior to EAUS in most studies [17, 23]. However, one prospective comparative study has shown that 3D-EAUS and MRI both were equally accurate in detecting anal fistulae [16].

Although 3D-EAUS was not accurate in some of the fistulae, it was able to give useful information about the sphincter defects which is important in planning the surgery in order to minimise damage to the anal sphincter complex. Those with fistulae with minimal sphincter involvement in 3D-EAUS and without sphincter defects can be safely laid open and allowed to granulate and heal. Those with fistulae with considerable sphincter involvement in 3D-EAUS without associated collection or pararectal cavities need staged seton fistulotomy. Deeper collections detected during 3D-EAUS may need a drainage seton with or without an irrigation catheter before performing a definitive seton fistulotomy. Therefore, it may help the surgeon to choose the appropriate surgical intervention.

In our setting, 3D-EAUS accurately predicted the characteristics of fistula-in-ano in 70.9% of patients with the highest accuracy for transphincteric fistulae (87%) while the accuracy was variable in other types. Therefore, 3D-EAUS had a good accuracy in selected types of fistulae and particularly useful in identifying sphincter defects before surgery.

## Limitations

Our study is a retrospective analysis of a small cohort of patients. We used surgical findings as the reference standard as other modes of imaging such as pelvic MRI was not routinely available in our unit.

## Additional file

**Additional file 1: Annexure S1.** Diagram used in the study to record the surgical findings.

## Abbreviations

3D EAUS: three-dimensional endoanal ultrasound scan
EUA: examination under anaesthesia
MRI: magnetic resonance imaging
