# Supplementary material for: Value of three-dimensional endoanal ultrasound scan (3D-EAUS) in preoperative assessment of fistula-in-ano
Source: BMC Res Notes. 2019 Jan 29;12:66. doi: 10.1186/s13104-019-4098-2 (PMC6352344; doi:10.1186/s13104-019-4098-2)
Supplement: Supplementary file 1 — Additional file 1: Annexure S1. Diagram used in the study to record the surgical findings. [file 13104_2019_4098_MOESM1_ESM.pdf]

Fistula Operation Notes

Patient Details

Name -

Age - Gender -

Address - Telephone No. -

Clinic No. (Preferred) - BHT No. - Ward

Description of Fistula (Please circle)

1. PRIMARY TRACT (Please circle)
    a. Superficial
    b. Inter-sphincteric
    c. Trans-sphincteric
    d. Supra-sphincteric
    e. Extra-sphincteric
2. INTERNAL OPENING (Please fill)
    a. Number -
    b. Site ____________ o'clock
    c. Level – (Please circle)
        i. Below
        ii. At
        iii. Above
        (i–iii: the Dentate Line)
        iv. Rectum
3. EXTERNAL OPENING (Please fill)
    a. Number -
    b. Site ____________ o'clock
4. HORSE SHOEING (Please circle)
    a. Inter-sphincteric
    b. Intra-levator (in ischiorectal fossa)
    c. Supra-levator
5. ABSCESS (Please circle)
    a. Superficial
    b. Inter-sphincteric
    c. Intra-levator (in ischiorectal fossa)
    d. Supra-levator
6. OTHER ANAL CONDITIONS (Please circle)
    a. Fissure
    b. Haemorrhoids
    c. Other

(SUMMARY OF TREATMENT – see over)

Anterior

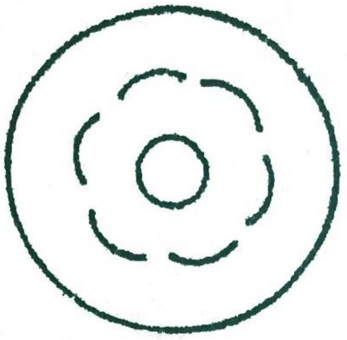

Right **Left**

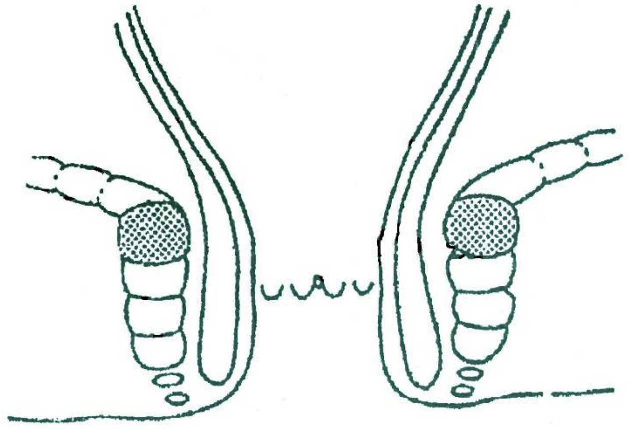

Anterior

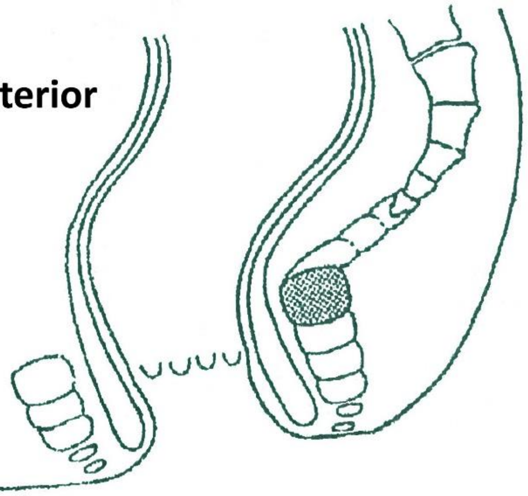

SUMMARY OF TREATMENT

No.	
	Date - Surgeon - Anaesthetist -<br>Procedure -<br>Notes -
	Date - Surgeon - Anaesthetist -<br>Procedure -<br>Notes -
	Date - Surgeon - Anaesthetist -<br>Procedure -<br>Notes -
